# UVB-mediated DNA damage induces matrix metalloproteinases to promote photoaging in an AhR- and SP1-dependent manner

**DOI:** 10.1172/jci.insight.156344

**Published:** 2022-05-09

**Authors:** Daniel J. Kim, Akiko Iwasaki, Anna L. Chien, Sewon Kang

**Affiliations:** 1 Department of Immunobiology, Yale University School of Medicine, New Haven, Connecticut, USA.; 2 Howard Hughes Medical Institute, Chevy Chase, Maryland, USA.; 3 Department of Dermatology, Johns Hopkins Medicine, Baltimore, Maryland, USA.

**Keywords:** Aging, Dermatology, Collagens, Skin, Transcription

## Abstract

It is currently thought that UVB radiation drives photoaging of the skin primarily by generating ROS. In this model, ROS purportedly activates **activator protein-1** to upregulate MMPs 1, 3, and 9, which then degrade collagen and other extracellular matrix components to produce wrinkles. However, these MMPs are expressed at relatively low levels and correlate poorly with wrinkles, suggesting that another mechanism distinct from ROS and MMP1/3/9 may be more directly associated with photoaging. Here we show that MMP2, which degrades type IV collagen, is abundantly expressed in human skin, increases with age in sun-exposed skin, and correlates robustly with aryl hydrocarbon receptor (AhR), a transcription factor directly activated by UV-generated photometabolites. Through mechanistic studies with HaCaT human immortalized keratinocytes, we found that AhR, specificity protein 1 (SP1), and other pathways associated with DNA damage are required for the induction of both MMP2 and MMP11 (another MMP implicated in photoaging), but not MMP1/3. Last, we found that topical treatment with AhR antagonists vitamin B_12_ and folic acid ameliorated UVB-induced wrinkle formation in mice while dampening MMP2 expression in the skin. These results directly implicate DNA damage in photoaging and reveal AhR as a potential target for preventing wrinkles.

## Introduction

UV radiation is a ubiquitous environmental carcinogen that induces numerous harmful effects in the skin. Comprising 95% of the photons that reach the Earth’s surface (1), UVA radiation (range 320–400 nm) generates ROS in both the dermis and epidermis, leading to the activation of various oxidative stress pathways. The MMPs that are upregulated as a result are thought to drive extrinsic aging (or photoaging) of the skin, degrading type I and type III collagen in the dermis to form fine and coarse wrinkles (2, 3). UVB radiation (range 280–320 nm) is another important component of UV primarily penetrating the epidermal layers, which not only contributes ROS to the skin like UVA, but also directly damages DNA, characteristically introducing bulky adducts like cyclobutane pyrimidine dimers (CPDs) and double-stranded breaks (DSBs) (4). While the central role of DNA damage is already readily appreciated in skin carcinogenesis (5), it is still unclear whether UVB-induced DNA damage holds any nonredundant roles in inducing MMP expression and driving photoaging.

Among the MMP genes that are induced by UV, MMP1, MMP3, and MMP9 are often considered to be the primary mediators of photoaging. It is thought that UV-induced ROS activates MAPK pathways that subsequently activate activator protein-1 (AP-1) to upregulate MMP1, MMP3, and MMP9 (6). Conversely, retinoids are believed to reverse the effects of photoaging — clinically effacing wrinkles — by transrepressing the AP-1 pathway (7), thereby suppressing MMP expression and restoring lost collagen (8, 9). The apparent absence of AP-1 binding sites in the proximal promoter regions of other MMP genes has further honed the field’s intense focus on this trio of MMPs. There is some evidence, however, that the role of these MMPs in the skin may be overstated, particularly in the case of MMP1 and MMP3. In addition to MMP1/3/9 only being variably expressed across melanoma cell lines (10), type I collagen degradation activity (associated with MMP1) and MMP3 levels are not elevated in UV-exposed, wrinkle-bearing skin (11), raising the question of which MMPs are more relevant for photoaging.

MMP2, encoding for 72 kDa gelatinase, represents a promising candidate. Induced by UVB irradiation in human skin (12), MMP2 targets type IV collagen present in the dermal-epidermal junction, much like MMP9, and is elevated in wrinkle-bearing skin (11). Moreover, MMP2 is uniformly elevated across melanoma cell lines and has also been associated with more invasive cancers and poorer prognoses (10). Despite these clinical associations, however, it is still unknown exactly how UV induces MMP2 expression.

Alongside the generation of ROS in the skin, UV produces various aromatic photometabolites (most notably 6-formylindolo[3,2-b]carbazole, FICZ) that directly bind and agonize the aryl hydrocarbon receptor (AhR) (13). Upon activation, AhR localizes to the nucleus, heterodimerizes with aryl hydrocarbon receptor nuclear translocator (ARNT), and binds xenobiotic response elements (XREs) to upregulate classic target genes like *Cyp1b1* (14). As part of its pleiotropic transcriptional response, AhR regulates a number of dermatologic processes including the tanning response (15) and maintenance of barrier immunity (16). Furthermore, past studies have demonstrated that treatment of melanoma cells with the AhR agonist 2,3,7,8-tetrachlorodibenzodioxin (TCDD) is sufficient to induce expression of MMP2 (17). In light of these studies, we hypothesized that UVB may be inducing MMP2 in an AhR-dependent fashion in keratinocytes.

In the present study, we unexpectedly found that AhR is required, but not sufficient, for UVB-mediated induction of MMP2 in HaCaT human keratinocytes. Through a series of mechanistic studies, we found that UVB-mediated induction of MMP2 and MMP11 (another MMP previously implicated in photoaging) requires the transcriptional factor SP1, DNA damage sensor ataxia telangiectasia mutated (ATM), and p38/JNK MAPK pathways. We further discovered that chemically induced DNA damage was sufficient to induce MMP2/11 but not MMP1/3. Underscoring the clinical relevance of this new model, topical treatment with vitamin B_12_ or folic acid (FA) (naturally occurring antagonists of AhR) significantly ameliorated UVB-induced wrinkles and sunburns, while reducing epidermal expression of MMP2. Therefore, by potentially linking DNA damage to MMP expression, this AhR- and SP1-mediated mechanism not only expands the field of photoaging to include other potentially more relevant MMPs, but also provides, to our knowledge, novel molecular targets for treating symptoms of photoaging.

## Results

### Mmp2 and Mmp11 correlate with age, sun exposure, and AhR pathways in human skin.

**Figure 1:**
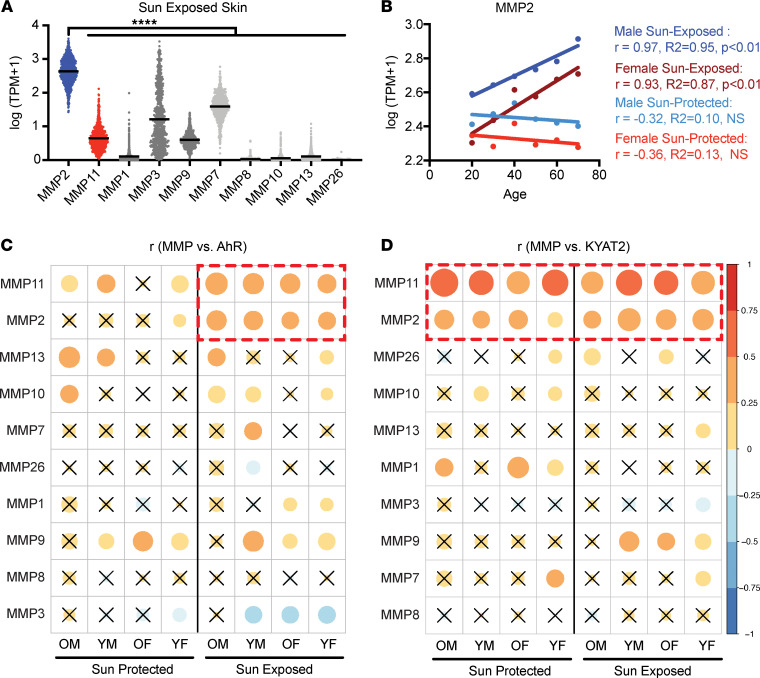
*Mmp2* mRNA expression increases with age in sun-exposed human skin and correlates with AhR pathway expression. (**A**) MMP mRNA expression in sun-exposed human skin. (**B**) *Mmp2* mRNA expression with increasing age in human skin. Correlations between MMP expression and (**C**) *Ahr* and (**D**) *Kyat2* mRNA in human skin across donor demographics (OM, older males; YM, younger males; OF, older females; YF, younger females). Color represents correlation coefficients. Size represents *P* values. Crossed-out circles indicate nonsignificant correlations (*P* > 0.05). All data are log_10_-transformed TPM values from GTEx. *n* = 234 male sun-exposed samples, 467 female sun-exposed samples, 193 male sun-protected samples, and 411 female sun-protected samples. *P* values were calculated by Pearson’s correlation, unpaired 2-tailed Mann-Whitney test, or 1-way ANOVA; ****P < 0.0001.

To begin assessing whether MMP2 might be a relevant mediator of photoaging, we first measured its mRNA expression in normal human skin using the Genome-Tissue Expression (GTEx) RNA-Seq data set. In addition to being the most abundantly expressed photoaging-associated MMP gene (6) (Figure 1A), *Mmp2* mRNA steadily increased with age in both male and female skin, but only in sun-exposed skin (Figure 1B and Supplemental Figure 1, A and B; supplemental material available online with this article; https://doi.org/10.1172/jci.insight.156344DS1). This pattern stands in stark contrast to the expression profiles of other MMP genes, which either did not have a meaningful relationship with age, as in the case of *Mmp1* (Supplemental Figure 1C), or even decreased with age, as with *Mmp3* (Supplemental Figure 1D). Although *Mmp9* expression increased with age (Supplemental Figure 1E), it did not exhibit as dramatic a difference as did *Mmp2* between sun-exposed and sun-protected skin (Figure 1B).

Next, given prior reports that AhR activation induces MMP2 in melanoma (17), we evaluated whether it might correlate with *Mmp2* expression in these skin samples. We stratified samples by donor demographics (using both age and sex) and generated a correlation map comparing *Ahr* expression with MMPs that have been implicated previously in photoaging (6). Only *Mmp2* and *Mmp11* positively correlated with *Ahr* expression across all demographics and exclusively in sun-exposed skin (Figure 1C), which may reflect its elevated content of AhR ligands from UVB-induced photometabolites like FICZ (13). To determine how MMPs might correlate with endogenous AhR ligands (which should be at comparable levels in sun-exposed and sun-protected skin), we repeated this analysis by comparing MMP expression with *Kyat2*, which is involved in the generation of kynurenic acid, a major endogenous ligand responsible for basal stimulation of the AhR pathway (18). As might be expected, *Kyat2* expression positively correlated with *Mmp2* and *Mmp11* mRNA — but not other evaluated genes — across all demographics, in both sun-protected and sun-exposed skin (Figure 1D). This clustering of *Mmp2* and *Mmp11* suggests that both MMP genes could be regulated by the same AhR-dependent pathway. Consistent with this idea, the expression of *Mmp11* also steadily increased with age in sun-exposed skin (Supplemental Figure 1F), similar to *Mmp2* but unlike *Mmp1* and *Mmp3*. Therefore, we next characterized the UV-induced transcriptional response of both *Mmp2* and *Mmp11*.

### AhR is required, but not sufficient, for UVB-induced MMP2 and MMP11 expression.

**Figure 2:**
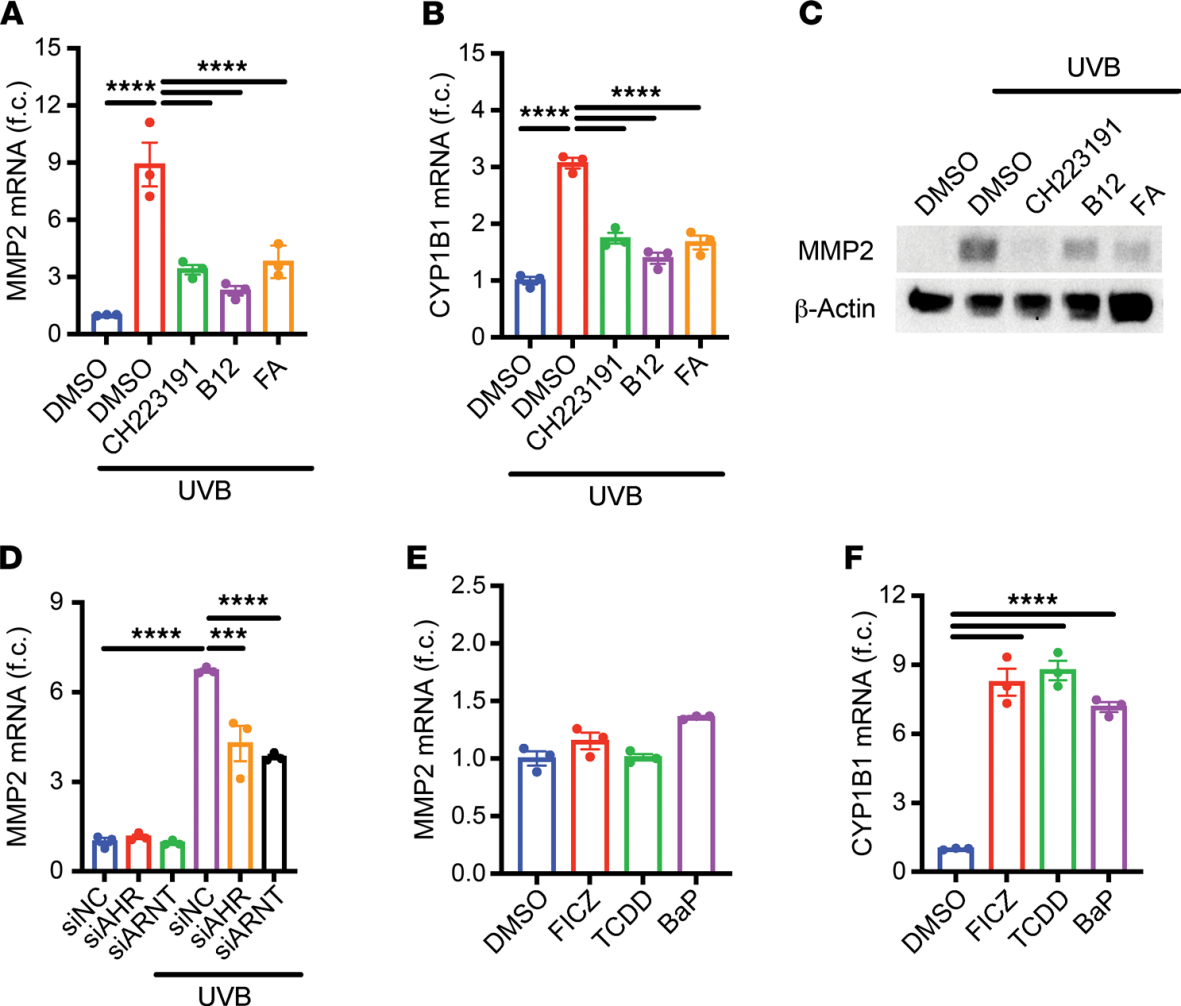
AhR is required, but not sufficient, for UVB-mediated induction of *Mmp2*. (**A**–**C**) HaCaT keratinocytes were irradiated with 200 mJ/cm^2^ UVB and subsequently treated with DMSO, 10 μM AhR antagonist CH223191, 50 ng/mL vitamin B_12_, or 20 ng/mL FA for 24 hours. (**A**) *Mmp2* and (**B**) *Cyp1b1* mRNA expression were measured by RT-qPCR and normalized to *Hprt1* and DMSO-treated samples (*n* = 3). (**C**) Protein levels of MMP2 and β-Actin were measured by Western blot. (**D**) HaCaT keratinocytes were transfected with negative control siRNA (siNC), siAHR, or siARNT and subsequently irradiated with 100 mJ/cm^2^ UVB. Cells were lysed for analysis 24 hours later. *Mmp2* mRNA was measured by RT-qPCR and normalized to *Hprt1* and siNC-transfected samples (*n* = 3). (**E** and **F**) HaCaT keratinocytes were treated with DMSO, 10 nM FICZ, 5 nM TCDD, or 5 μM BaP for 24 hours. (**E**) *Mmp2* and (**F**) *Cyp1b1* mRNA were measured by RT-qPCR and normalized to *Hprt1* and DMSO-treated samples (*n* = 3). Data are means ± SEM. *P* values were calculated by Pearson’s correlation or 1-way ANOVA; ***P < 0.001, ****P < 0.0001.

To assess whether AhR is required for UVB-mediated induction of *Mmp2* and *Mmp11*, we irradiated HaCaT keratinocytes with a single dose of UVB at 200 mJ/cm^2^ — equivalent to approximately 20 minutes of sunlight on a clear summer day in Europe (19) — and then treated them with synthetic AhR antagonist CH-223191 or naturally occurring AhR antagonists vitamin B_12_ or FA (20). This treatment abrogated the UVB-mediated induction of *Mmp2* and *Mmp11* (Figure 2A and Supplemental Figure 2A) and suppressed *Cyp1b1* mRNA (Figure 2B), confirming inhibition of AhR activity. Protein levels of MMP2 largely followed changes in its mRNA expression in both HaCaT keratinocytes (Figure 2C) and primary human dermal fibroblasts (Supplemental Figure 2B).

To confirm the requirement of AhR through a genetic approach, we knocked down AhR and its canonical heterodimerization partner ARNT with siRNA (Supplemental Figure 2C) and found that this significantly attenuated the induction of *Mmp2* and *Mmp11* mRNA (Figure 2D and Supplemental Figure 2, D and E) and protein (Supplemental Figure 2F). Knockdown of AhR did not alter the mRNA expression of *Mmp1* or *Mmp3* (Supplemental Figure 2, G and H) but significantly reduced that of *Mmp9* (Supplemental Figure 2I). These data suggest that, first, *Mmp2* and *Mmp11* are regulated by a distinct AhR-dependent transcriptional mechanism and, second, that *Mmp9* may be coregulated by this new AhR-dependent mechanism and transcription factor AP-1 (which also regulates *Mmp1* and *Mmp3*).

Next, we investigated whether AhR activation is sufficient to induce *Mmp2* and *Mmp11* mRNA in HaCaT cells. Unexpectedly, treatment with 3 prototypical AhR agonists — TCDD, FICZ, and benzo(a)pyrene (BaP) — did not result in induction of *Mmp2* and *Mmp11* (Figure 2E and Supplemental Figure 2J), despite strong upregulation of *Cyp1b1* (Figure 2F). This suggested there might be another pathway activated by UVB that is working with AhR to induce *Mmp2* and *Mmp11* mRNA. Consistent with this idea, treatment with TCDD in the presence of UVB irradiation further induced *Mmp2* and *Mmp11* mRNA (Supplemental Figure 2, K and L), despite no further changes in *Cyp1b1* mRNA (Supplemental Figure 2M), indicating that *Mmp2* and *Mmp11* are not induced simply as AhR target genes.

### SP1 is required for UVB-mediated MMP2/11 expression and acts downstream of AhR.

**Figure 3:**
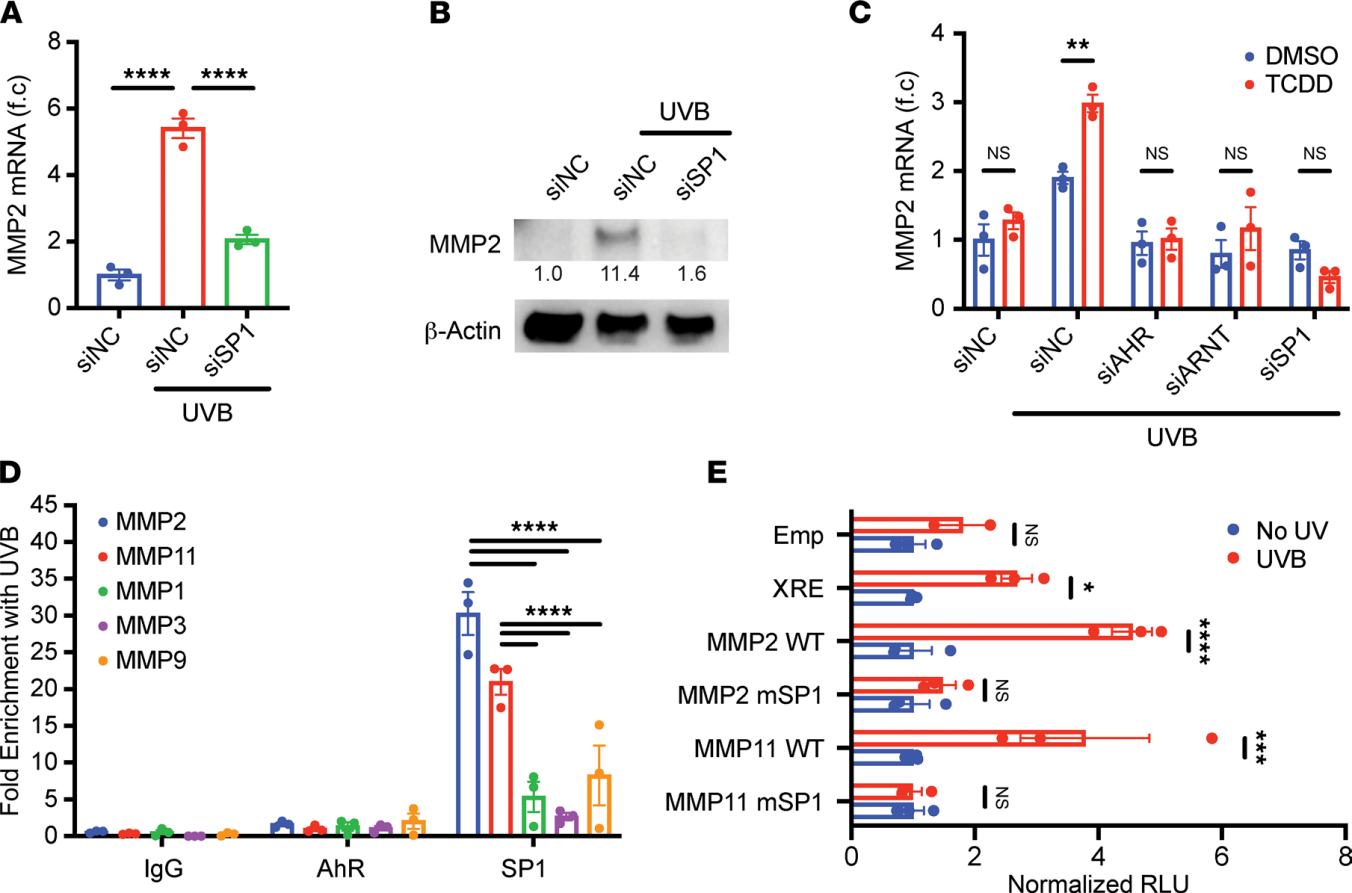
SP1 binding to promoter region is required for UVB-mediated induction of *Mmp2* and *Mmp11*. (**A** and **B**) HaCaT keratinocytes were transfected with siNC or siSP1 and irradiated with 100 mJ/cm^2^ UVB. Cells were lysed 24 hours later for analysis. (**A**) *Mmp2* mRNA was measured by RT-qPCR and normalized to *Hprt1* and siNC-transfected samples (*n* = 3). (**B**) Protein levels of MMP2 and β-Actin were measured by Western blot. Relative densitometry measurements are shown for MMP2. (**C**) HaCaT keratinocytes were transfected with indicated siRNA, irradiated with UVB, and subsequently treated with DMSO or 25 nM TCDD. Cells were lysed 24 hours later for analysis. *Mmp2* mRNA was measured by RT-qPCR and normalized to *Hprt1* (*n* = 3). (**D**) HaCaT cells were irradiated with UVB and lysed 24 hours later. Enrichment of AhR and SP1 at MMP promoter regions was measured by ChIP-PCR, normalizing to input samples and nonirradiated samples (*n* = 3). (**E**) HaCaT cells were cotransfected with Renilla luciferase construct and one of the following: an empty firefly luciferase construct (Emp), a firefly luciferase construct containing AhR response elements (XRE), WT MMP2 promoter (MMP2 WT), SP1 binding site mutant MMP2 promoter (MMP2 mSP1), WT MMP11 promoter (MMP11 WT), or SP1 binding site mutant MMP11 promoter (MMP11 mSP1). Cells were irradiated with UVB and lysed 24 hours later. Fold induction of relative luciferase units (RLU) was calculated by normalizing firefly luciferase activity to Renilla luciferase activity and nonirradiated samples (*n* = 3). Data are means ± SEM. *P* values were calculated by 1-way ANOVA or 2-way ANOVA. *P < 0.05, **P < 0.01, ***P < 0.001, ****P < 0.0001.

SP1 is a ubiquitous transcriptional factor that has previously been shown to regulate the expression of both MMP2 and MMP11 in nonskin cells (21, 22). Given the role of SP1 in responding to DNA damage (23, 24) — which would be present in both UVB-irradiated cells and cancer — we hypothesized that SP1 might directly mediate the induction of *Mmp2* and *Mmp11* mRNA by UVB. We found that knocking down SP1 with siRNA (Supplemental Figure 3A) abrogated the induction of *Mmp2* and *Mmp11* mRNA (Figure 3A and Supplemental Figure 3B) and the MMP2 protein (Figure 3B). Interestingly, as with AhR, SP1 knockdown did not suppress the induction of *Mmp1* or *Mmp3* (Supplemental Figure 3, C and D) but attenuated *Mmp9* mRNA induction (Supplemental Figure 3E). *Cyp1b1* mRNA was unchanged (Supplemental Figure 3F) with SP1 silencing, suggesting that SP1 might act downstream of AhR. Consistent with this idea, the knockdown of SP1, alongside the knockdowns of AhR and ARNT, abrogated the induction of *Mmp2* and *Mmp11* by TCDD in the context of UVB irradiation (Figure 3C and Supplemental Figure 3G), confirming that the effect of AhR on irradiated cells is mediated by SP1.

Given this requirement for SP1, we next investigated whether SP1 binds directly to the promoter regions of *Mmp2* and *Mmp11* with UVB irradiation. ChIP-PCR revealed that SP1, but not AhR, was significantly more enriched with irradiation in the promoter regions of *Mmp2* and *Mmp11* than in those of *Mmp1*, *Mmp3*, and *Mmp9* (Figure 3D). Interestingly, in addition to each promoter containing at least 1 validated SP1 binding site (which are heavily composed of G and C nucleotides) (21, 22), the 200 bp promoter regions of *Mmp2* and *Mmp11*, as a whole, feature an overall elevated guanosine-cytosine (GC) content (68% and 75%, respectively), which could represent a potential mechanism by which SP1 responsiveness is achieved via selection. Notably, this GC content was similar to that in the promoter region of another SP1 target gene *Vegfa* (74%) (Supplemental Figure 3, H and I) (25). In contrast, promoters of other evaluated MMP genes did not exceed 50% GC content (as might be expected by chance), whereas the MMP9 promoter had a more intermediate GC content (59%, Supplemental Figure 3, H and I). Finally, through mutation analysis of luciferase constructs containing the promoter regions of human *Mmp2* and *Mmp11*, we found that the abrogation of validated SP1 binding sites in these 2 promoters eliminated their responsiveness to UVB irradiation (Figure 3E). In sum, these results show that induction of *Mmp2* and *Mmp11* requires binding of SP1 to their promoters.

### DNA damage and MAPK pathways mediate induction of MMP2/11.

**Figure 4:**
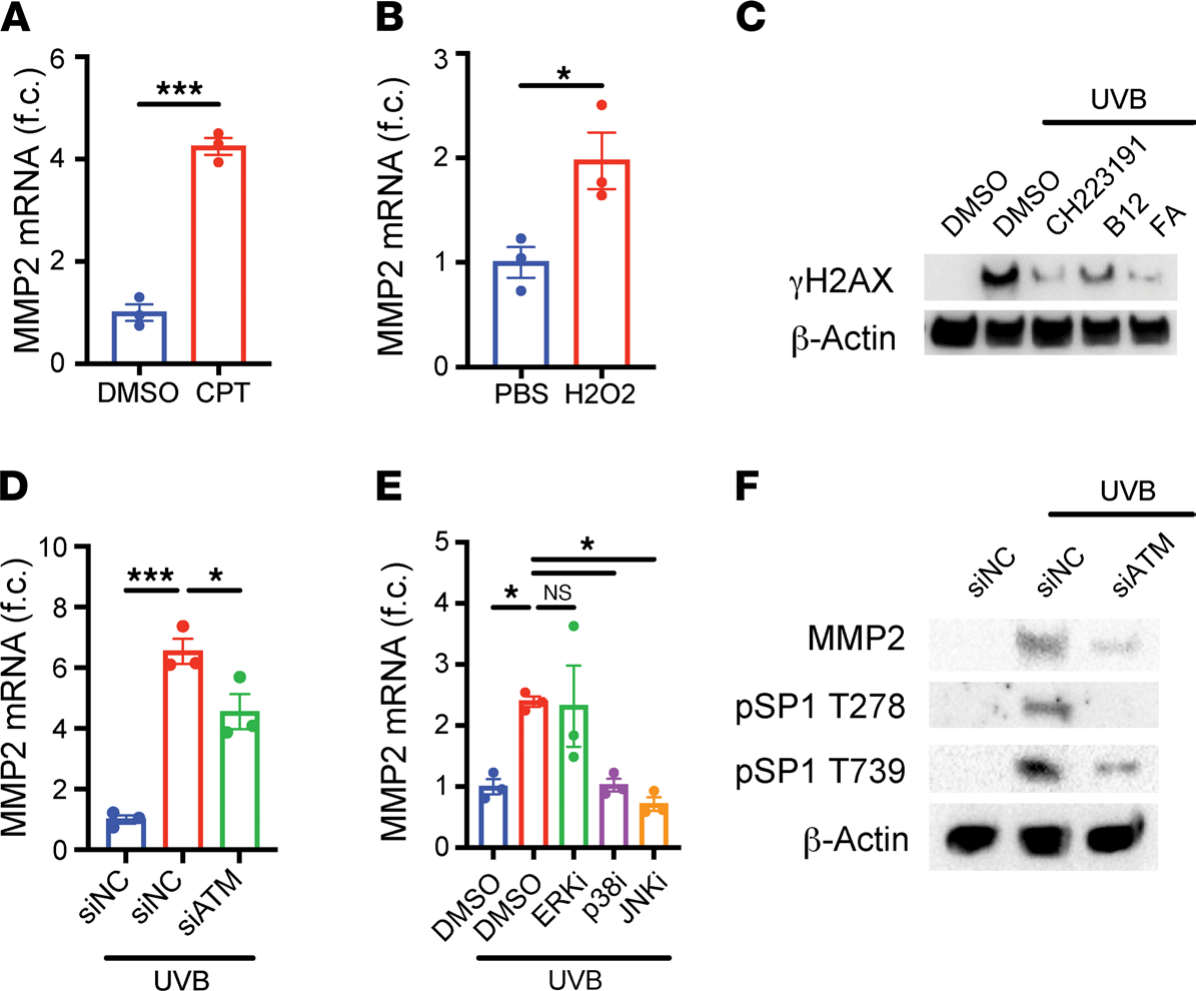
DNA damage, ATM, p38, and JNK are implicated in the induction of *Mmp2* mRNA. (**A**) HaCaT keratinocytes were treated with DMSO or 5 μM camptothecin (CPT) for 24 hours. *Mmp2* mRNA was measured by RT-qPCR and normalized to *Hprt1* and DMSO-treated samples (*n* = 3). (**B**) HaCaT keratinocytes were treated with PBS or 1 mM H_2_O_2_ for 24 hours. *Mmp2* mRNA was measured by RT-qPCR and normalized to *Hprt1* and PBS-treated samples (*n* = 3). (**C**) HaCaT keratinocytes were irradiated with 200 mJ/cm^2^ UVB and subsequently treated with DMSO, 10 μM CH-223191, 50 ng/mL vitamin B_12_, or 20 ng/mL FA for 24 hours. Protein levels of γH2AX and β-Actin were measured by Western blot. (**D** and **F**) HaCaT keratinocytes were transfected with siNC or siATM and irradiated with UVB. Cells were lysed 24 hours later for analysis. (**D**) *Mmp2* mRNA was measured by RT-qPCR and normalized to *Hprt1* and siNC-transfected samples (*n* = 3). (**F**) Protein levels of MMP2, phosphorylated SP1 (Thr-278 and Thr-739 residues), and β-Actin were measured by Western blot. (**E**) HaCaT keratinocytes were irradiated with UVB and treated with DMSO, 50 μM PD98059 (ERK inhibitor), 10 μM SB203580 (p38 inhibitor), or 25 μM SP600125 (JNK inhibitor) for 24 hours. *Mmp2* mRNA was measured by RT-qPCR and normalized to *Hprt1* and DMSO-treated samples (*n* = 3). Data are means ± SEM. *P* values were calculated by unpaired 2-tailed Student’s *t* test and 1-way ANOVA. *P < 0.05, ***P < 0.001.

Next, we sought to identify how UVB might activate SP1. As mentioned previously, SP1 is characteristically activated with DNA damage, especially with DSBs (24, 26). DSBs can arise indirectly with UVB irradiation when actively dividing cells like basal keratinocytes attempt to replicate DNA containing bulky photoproducts CPDs that are not properly removed by nucleotide excision repair (NER) (27–30). To evaluate whether DNA damage drives UVB-mediated induction of *Mmp2* and *Mmp11*, we treated HaCaT cells with either camptothecin (a topoisomerase I inhibitor that induces single-stranded breaks that can progress to DSBs; ref. 31) or hydrogen peroxide (which causes oxidative damage to DNA that can progress to DSBs; ref. 32). Both compounds were sufficient to induce mRNA of *Mmp2* and *Mmp11* (Figure 4, A and B; and Supplemental Figure 4, A and E), but not *Mmp1* or *Mmp3* mRNA (Supplemental Figure 4, B, C, F, and G) — resembling the dichotomous patterns seen with AhR and SP1 knockdown experiments. Interestingly, *Mmp9* mRNA was inducible with hydrogen peroxide (Supplemental Figure 4H), but not with camptothecin (Supplemental Figure 4D).

Given the role of AhR in suppressing NER and clearance of CPDs (33), we hypothesized that AhR activation by UVB could promote DSB accumulation (as a result of persistent CPDs), ultimately leading to SP1 activation and induction of *Mmp2* and *Mmp11* mRNA. Such a model would help explain why AhR activation alone, in the absence of CPD generation by UVB, is not sufficient for inducing *Mmp2* and *Mmp11* but may still be required for MMP induction in the presence of CPDs. Consistent with this hypothesis, we found that treatment with small-molecule inhibitors of AhR decreased the abundance of phosphorylated H2AX, a common marker of DSBs (Figure 4C). Furthermore, knockdown of the serine/threonine protein kinase ATM (Supplemental Figure 4I), a classic sensor of DSBs, attenuated the induction of *Mmp2* and *Mmp11* mRNA in irradiated keratinocytes (Figure 4D and Supplemental Figure 4J). This requirement for ATM — alongside the requirements for AhR, ARNT, and SP1 — was replicated in normal primary human dermal fibroblasts (Supplemental Figure 4, K and L), suggesting that this model may be broadly relevant across the different cell types of the skin and in more primary, p53-sufficient contexts.

Next, we examined how UVB-mediated DNA damage might ultimately activate SP1. Classically associated with stress responses, p38 and JNK MAPK pathways are activated by both UVB (34) and DNA damage (35) and have previously been shown to induce transcription of genes via SP1 phosphorylation (36, 37). Targeting the 3 major MAPK pathways with small-molecule inhibitors, we found that inhibition of p38 and JNK, but not ERK, abrogated the induction of *Mmp2* and *Mmp11* mRNA (Figure 4E and Supplemental Figure 4M). These results are consistent with prior reports that anisomycin, a small molecule activator of p38 and JNK pathways, is sufficient to induce *Mmp2* mRNA (38). Next, we found that knockdown of ATM, which acts upstream of MAPK pathways (39), decreased phosphorylation of SP1 at Thr-278 and Thr-739 (Figure 4F), residues that are targeted by p38 and JNK pathways and also known to induce the transcriptional activity of SP1 (36, 37). These results implicate MAPK pathways and SP1 phosphorylation as possible downstream mediators of UVB irradiation.

### Treatment with vitamin B_12_ and FA attenuates UVB-associated sunburn and wrinkles in mice.

Last, to assess the clinical relevance of this new AhR-dependent mechanism, we irradiated mice with increasing doses of UVB over a period of 16 weeks and treated them topically with either B_12_ or FA, immediately after each exposure. Sunburns are often associated with sun-induced DNA damage as they represent cell death resulting from excessive UVB irradiation (40). We first hypothesized that B_12_ and FA treatment, by antagonizing AhR, might ameliorate the severity of sunburns — presumably by rescuing NER activity, decreasing persistent DNA damage, and ultimately preventing cell death. Supporting this hypothesis, at week 10, irradiated mice exhibited more severe erythema and scaling than did nonirradiated or vitamin-treated mice (Supplemental Figure 5A). Through the use of a skin probe, we verified that treatments with B_12_ and FA not only reduced erythema but also decreased transepidermal water loss (TEWL) associated with UVB (Supplemental Figure 5, B and C).

**Figure 5:**
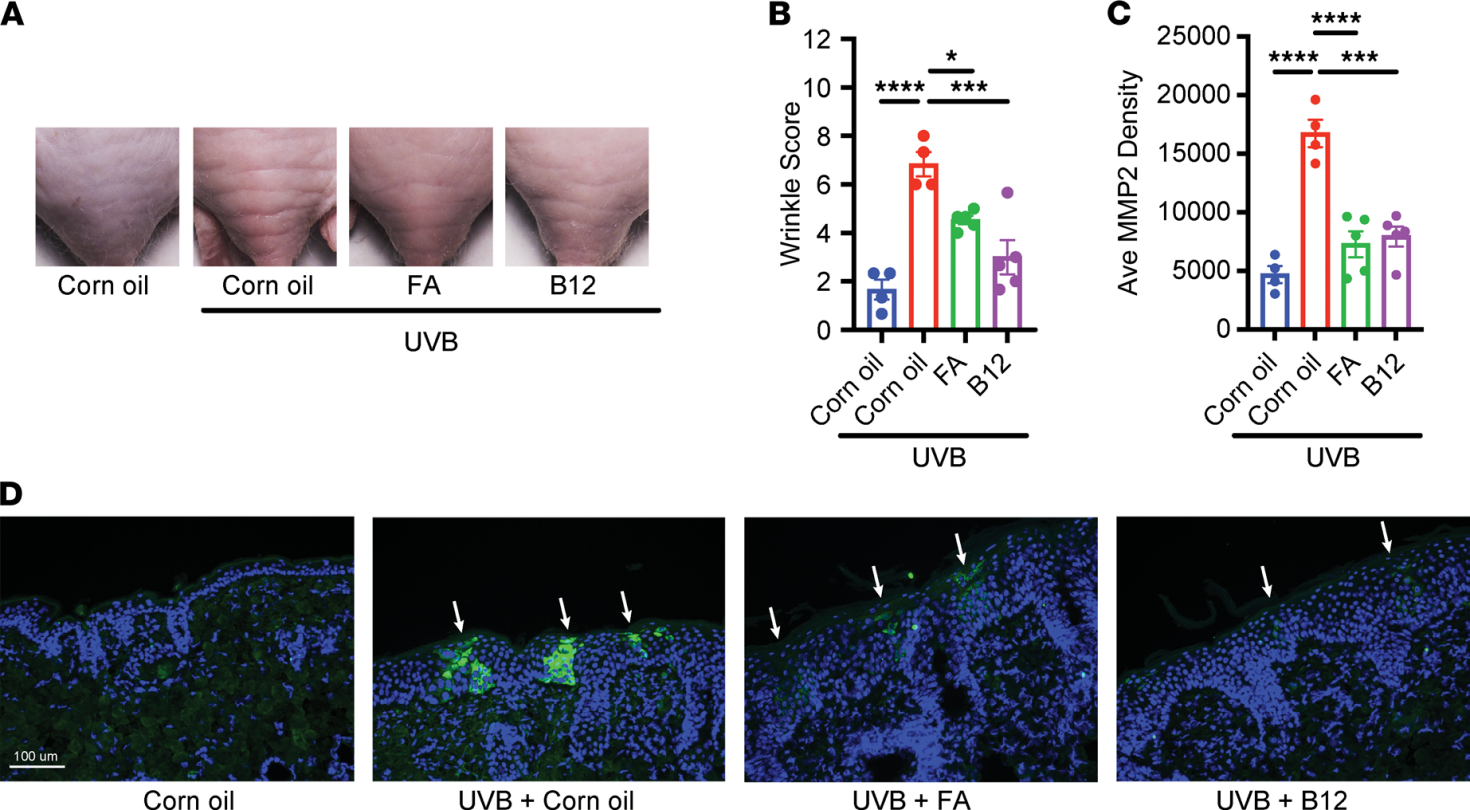
Treatment with vitamin B_12_ or FA ameliorates UVB-induced wrinkles and MMP2 expression. SKH1 hairless mice were irradiated 3 times a week for 16 weeks with gradually increasing doses. After each irradiation, corn oil, B_12_, or FA was topically applied to the dorsum of mice. At week 13, mice were evaluated for wrinkle formation. (**A**) Representative images of wrinkles from the lower dorsum of mice. (**B**) Wrinkles were graded by blinded investigators using the scale provided by Inomata et al. (11). (**C** and **D**) Sections perpendicular to the wrinkles were paraffin-fixed and stained with DAPI and anti-MMP2 antibody with fluorescence IHC. Bands of MMP2 expression for each section were identified at 200× original magnification, and fluorescence was quantified by ImageJ (NIH). (**C**) Average fluorescence intensity of MMP2 bands by mouse. (**D**) Representative images of MMP2 bands (white arrows) found on IHC (200× original magnification). Blue indicates DAPI staining; green indicates MMP2 staining. *n* = 4–5 mice for each treatment group. Data are means ± SEM. *P* values were calculated by 1-way ANOVA. *P < 0.05, ***P < 0.001, ****P < 0.0001.

We also hypothesized that the inhibition of AhR could attenuate wrinkle formation by suppressing MMP2 induction. At week 13, UVB-irradiated mice exhibited coarser wrinkles in the lower dorsum than did nonirradiated and vitamin-treated mice (Figure 5, A and B). Through fluorescence IHC of sections cut perpendicularly to the mice’s wrinkles, we observed that MMP2 expression localized as bright discrete bands in the epidermis (Figure 5D). Quantifying the fluorescence intensity of these bands, we found that B_12_- and FA-treated mice featured less intense bands than untreated UVB-irradiated mice (Figure 5C and Supplemental Figure 5D). Furthermore, the band intensities strongly correlated with wrinkle scores (Supplemental Figure 5E), indicating that these bands of MMP2 expression might be associated with wrinkles. In summary, these data suggest not only that MMP2 may play a critical role in wrinkle formation but also that vitamins B_12_ and FA may counteract photoaging by potentially suppressing AhR and MMP2.

## Discussion

**Figure 6:**
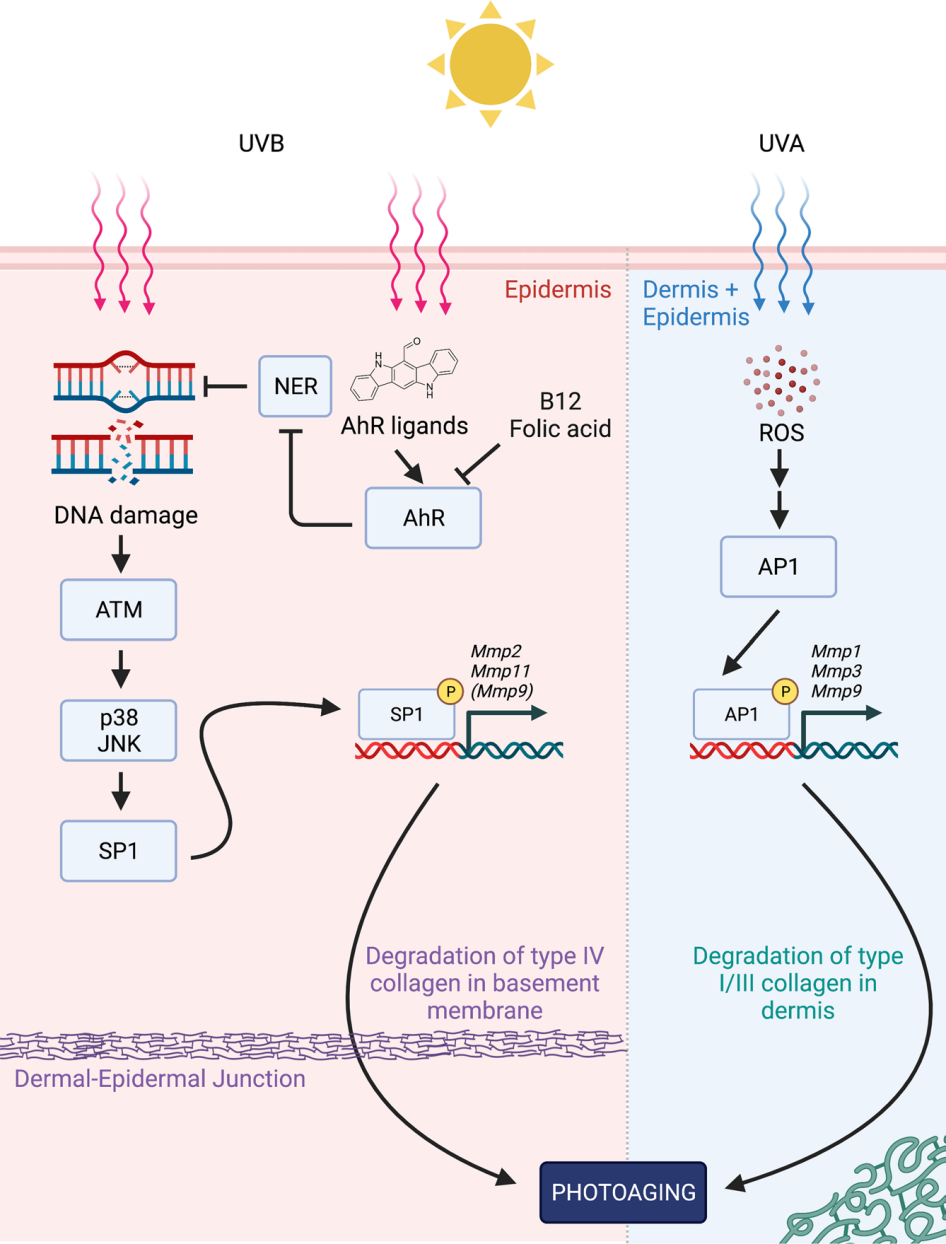
Proposed model for MMP induction in photoaging. In the epidermis, first, UVB-generated aromatic ligands activate AhR which suppress NER activity; second, enhanced DNA damage is detected by ATM kinase, which subsequently activates p38 and JNK MAPK pathways; and third, phosphorylated SP1 binds the GC-rich promoter regions of *Mmp2* and *Mmp11* (possibly alongside *Mmp9*) to promote their transcription. At the same time, in both the dermis and epidermis, UVA-generated ROS ultimately leads to the activation of AP1, leading to the upregulation of *Mmp1, Mmp3,* and *Mmp9* (previously shown). Both sets of MMP genes could then contribute to the signs of photoaging in skin with MMP2, primarily degrading type IV collagen in the basement membrane, and other MMPs degrading types I/III collagen in the dermis. By antagonizing AhR and promoting clearance of UVB-generated DNA damage, B_12_ and FA could potentially counteract photoaging. Diagram generated using BioRender.

With the current focus on ROS and MMP1/3/9 in the photoaging field, we demonstrate here that another major matrix metalloproteinase, MMP2 (alongside MMP11), is induced by UVB DNA damage through a distinct mechanism that relies on AhR, SP1, ATM, and p38/JNK MAPK (Figure 6). In brief, we propose first, that UVB irradiation causes DNA damage that is enhanced by AhR via suppression of NER; second, that ATM kinase senses the resulting DNA damage and subsequently activates p38 and JNK, which can phosphorylate SP1; and third, that activated SP1 binds to the promoter regions of *Mmp2* and *Mmp11* to induce their transcription. (*Mmp9* expression may be coregulated by both this mechanism and AP1.) Furthermore, based on in silico and in vivo studies, we propose that MMP2 may be a relevant driver in photoaging of the skin and that therapies aimed at reducing MMP2 (such as AhR antagonism by vitamin B_12_ and FA) can potentially counteract photoaging. What ultimately emerges from our work is a more comprehensive picture of photoaging in which both ROS and DNA damage resulting from UVB activate 2 distinct signaling pathways, leading to the induction of complementary batteries of MMP genes, which target both type I and type III collagen present in the dermis and type IV collagen present at the dermal-epidermal junction. With the inclusion of DNA damage, this new expanded model of photoaging also offers a more obvious link to carcinogenesis, whereby accumulating DNA damage in skin could conceivably lead to both wrinkles (by MMP induction) and cancer (which can later utilize MMPs to invade the underlying basement membrane).

While MMP2 was the primary focus of this paper and arguably the most promising candidate for photoaging given its expression pattern in human skin (Figure 1, A and B), we also found that *Mmp11* mRNA behaved similarly to *Mmp2* mRNA across all in vitro studies, indicating that there is a larger SP1 transcriptional program that is enacted with UVB. In addition to harboring validated SP1 binding sites, the proximal promoter regions of *Mmp2* and *Mmp11* were markedly elevated in their GC content, similar to another SP1 target *Vegfa* but in contrast to *Mmp1* and *Mmp3*. Regardless of whether this elevated GC content is merely the result of, or the means to, accumulating SP1 binding sites, it is compelling that MMP9 appears to have an intermediate GC content between MMP2/11 and MMP1/3. This finding, alongside the general observation that MMP9 clustered with MMP2 and MMP11 in most of our mechanistic studies, suggests that MMP9 could also be regulated by a similar SP1-mediated mechanism, perhaps alongside the currently accepted AP-1-mediated mechanism of induction. Curiously, recent work by Ujfaludi et al. (41) found that MMP9 did not cluster with MMP1 and MMP3 expression after UVB irradiation in a novel keratinocyte cell line HKerE6SFM, raising the possibility that AP-1 may not necessarily be the primary mode of regulation for *Mmp9* mRNA. Future work could further elucidate the role of SP1 and DNA damage in driving MMP9 induction.

We also learned from our studies that both natural and synthetic AhR inhibition attenuated double-stranded DNA damage resulting from 200 mJ/cm^2^ UVB irradiation (Figure 4C). Interestingly, it was reported earlier that, while AhR inhibition restores NER and clearance of CPDs, that same AhR inhibition actually introduces *more* DSBs in an NER-independent manner when keratinocytes are irradiated with 20 mJ/cm^2^ UVB (33), a significantly smaller dose than that used in the present study. Juxtaposed with our own experiments, then, we find it plausible that AhR inhibition may have opposing effects on DSBs — one mediated indirectly through enhanced CPD clearance, as suggested by work in NER-deficient XPB cells (42), and another that more directly promotes DSB, possibly via CHK1 inhibition (43). We further propose that these 2 effects are differentially highlighted depending on the UVB dose. At very low doses of UVB (when CPD burden is more modest, perhaps), the NER-restoring benefits of AhR antagonists may be limited, allowing the direct DSB-promoting effects to dominate. With higher doses of UVB (and many more CPDs that could progress to DSBs), however, the NER-restoring benefits of AhR inhibition may become more apparent and outweigh the direct DSB-promoting effects, leading to a net decrease in DSBs and H2AX phosphorylation. Consistent with this complex relationship between AhR and DSBs, we unexpectedly observed that the genetic knockdown of AhR and ARNT had a more modest suppression of *Mmp2* and *Mmp11* expression (Figure 2D and Supplemental Figure 2D) compared with small molecule inhibitors (Figure 2A and Supplemental Figure 2A), despite decreasing AhR activity substantially below baseline levels (Supplemental Figure 2E). Collectively, these results suggest that there is likely some intermediate level of AhR activity that optimally minimizes the net production of double-stranded DNA damage from UVB and the subsequent expression of MMP2/11.

Finally, our in vivo work with vitamin B_12_ and FA offer preliminary evidence that AhR antagonism could prove a useful clinical strategy in ameliorating some harmful effects of UV irradiation. We first observed that vitamin-treated mice exhibited less severe sunburns (Supplemental Figure 5, A–C), which is consistent with our model that AhR antagonism decreases UVB-induced DNA damage (Figure 4E). We also observed that the vitamin treatments improved the appearance of wrinkles and concomitantly decreased MMP2 expression in the epidermis. This finding could lend insight into how various natural extracts or even prenatal vitamins, which contain high levels of B_12_ or FA, might exert their long-purported (albeit often anecdotal) “anti-aging” effects. Additionally, given that vitamin B_12_ and FA are sensitive to photolysis (44, 45), topical delivery immediately after UV exposure (as in our mouse experiments) may prove a particularly effective strategy in directly repleting cutaneous stores of these vitamins and counteracting induction of MMPs.

Curiously, we observed that epidermal MMP2 was expressed in an almost binary manner, with sporadic “bands” (spanning all layers of the epidermis) in an “ON” state and the vast majority of cells in an “OFF” state (Figure 5D). While the epidermal expression of MMP2 has been described before in human and mouse skin (46–48), this particular “banding” pattern has not been previously described, to our knowledge. Especially considering that these IHC sections were cut perpendicularly to the wrinkles, the discrete, sporadic nature of these MMP2 bands and close association with wrinkle scores (Supplemental Figure 5E) provide strong correlative evidence that these bands could somehow spatially mirror the wrinkles themselves. Given prior work demonstrating that MMP2 is enzymatically active at the dermal-epidermal junction in UVB-induced wrinkles and degrading type IV collagen (11), we find it plausible that these MMP2 bands could represent longitudinal “fault lines” of sorts — with underlying compromise in structural integrity that could cause the skin to buckle upon mechanical shear. To be clear, this model of epidermal MMP2 forming wrinkles by targeting type IV collagen in the basement membrane is not mutually exclusive to the current model of UVA inducing MMPs in the dermis causing degradation of type I collagen. Rather, we see the 2 models as potentially synergistic with one another, degrading collagen at different layers of the skin and leading to the multifaceted changes characteristic of photoaging.

In conclusion, our data highlight MMP2 as a potential driver of UV-mediated photoaging, expanding the scope of the field beyond just ROS or MMP1/3/9. Implicating UVB-induced DNA damage, our proposed model not only complements the current model of photoaging that has heavily focused on ROS production but also provides exciting new avenues for rational drug design in the treatment of photoaging (Figure 6).

## Methods

### Cell lines, antibodies, and reagents.

HaCaT keratinocytes and normal human dermal fibroblasts (NHDF) were a gift from Luis Garza (Johns Hopkins University). Anti–β-actin (ab8227) and anti-MMP2 (WB: ab97779; IHC: ab86607) antibodies were purchased from Abcam. Anti-MMP11 antibody (NBP2-67670) was purchased from Novus. Anti–phospho-SP1 (Thr-278: PA5-106039; Thr-739: PA5-104771), HRP-conjugated anti–rabbit IgG (A27036), HRP-conjugated anti–mouse IgG (A28177), and A488-conjugated anti-mouse (A11029) antibodies were purchased from Invitrogen. Anti-AhR (83200S), anti-ARNT (5537S), anti-SP1 (9389S), anti-ATM (2873T), anti-γH2AX (2577S), and normal rabbit IgG (2729S) antibodies were purchased from Cell Signaling Technologies. CH223191 (C8124), vitamin B_12_ (V2876), and FA (F7876) were purchased from Sigma-Aldrich. PD98059 (9900S), SB203580 (5633S), and SP600125 (8177S) were purchased from Cell Signaling Technologies. The following siRNAs were purchased from Thermo Fisher: siNC (catalog 4390843), siAHR (catalog 4390824, s1199), siARNT (catalog 4392420, s1615), siSP1 (catalog 4392420, s13319), and siATM (catalog 4392420, s57221).

### GTEx analysis.

Transcripts per million (TPM) data from GTEx v8 were downloaded from https://gtexportal.org TPM counts for genes of interest were extracted using R and transformed into log_10_(TPM+1) values for further analysis. Expression analysis was performed with Microsoft Excel, GraphPad Prism, and R. The following packages were used in R: ggplot2, ggpubr, dplyr, ComplexHeatmap, CePa, corrplot, and RColorBrewer. For promoter GC content analysis, promoter regions of MMP genes were downloaded from the NCBI website and were analyzed by R.

### In vitro experiments.

Cells were grown at 37°C incubator at 5% CO_2_ in DMEM supplemented with 10% FBS and 1% penicillin-streptomycin. For all in vitro experiments, cells were plated with supplemented media and grown to 80% confluence. Cells were then serum-starved in MEM supplemented only with 1% penicillin-streptomycin for 48 hours. For siRNA knockdown experiments, siRNA was transfected with Lipofectamine RNAiMAX (Thermo Fisher Scientific, 13778075) at the start of serum starvation. For luciferase experiments, luciferase constructs were transfected using Lipofectamine 3000 (Thermo Fisher Scientific, L3000008) 24 hours after start of serum starvation. Prior to irradiation, serum-starved media were aspirated and wells were washed twice in prewarmed PBS. In a small volume of PBS, cells were then irradiated in ventilated cabinets equipped with UVB lamps at the indicated dose. Afterward, PBS was replaced with MEM supplemented with 10% FBS and 1% penicillin-streptomycin, and cells were returned to 37°C for 24 hours. Cells were then lysed for downstream analyses. All cellular experiments were repeated with at least 3 biological replicates.

### In vivo experiments.

Male 16-week-old SKH1 hairless mice (Charles River Laboratories) were irradiated 3 times a week in the aforementioned UVB cabinets, as described previously by Inomata et al. with slight modifications (11). Briefly, mice received 36 mJ/cm^2^ in the first week and were increased to 54, 72, 108, 144, 162, 180, and 198 mJ/cm^2^ at 1-week intervals. Mice were given 216 mJ/cm^2^ UVB at weeks 9 and 10 and 180 mJ/cm^2^ for all subsequent weeks. Immediately after each irradiation, mice were topically treated with 400 μL of corn oil, 250 μg/mL B_12_, or 100 μg/mL FA (dissolved in corn oil) across the entire dorsal surface. At week 10, mice were photographed and evaluated for sunburns. Erythema and TEWL were also quantified by DermaLab skin probes (Cortex Technologies). At week 13, mice were photographed and evaluated for wrinkles. Blinded investigators graded photographs of wrinkles on the lower dorsum of mice using the scale previously described by Inomata et al. (11). All mice were maintained under specific pathogen-free conditions at an American Association for the Accreditation of Laboratory Animal Care–accredited animal facility at Johns Hopkins University and were housed according to procedures described in the *Guide for the Care and Use of Laboratory Animals* (National Academies Press, 2011).

### IHC.

Mice were anesthetized 48 hours after the last irradiation at week 16. Formalin-fixed, paraffin-embedded sections were obtained from the lower dorsum of mice, oriented perpendicularly to the wrinkles. After deparaffinization using Trilogy buffer (Cell Marque), slides were washed 3 times with PBS and blocked with 10% normal goat serum diluted in PBS with 0.05% Tween 20 (PBST) for 1 hour. Samples were then incubated with primary antibody diluted in PBST (anti-MMP2, 1:200) overnight at 4°C. Following 3 washes with PBST on an orbital shaker, samples were incubated with a secondary antibody (goat anti-mouse-A488; diluted 1:1000 in PBST) for 1 hour at room temperature on an orbital shaker, protected from light. After 3 more washes with PBST, samples were stained with DAPI (diluted 1:1000 in PBS) for 2 minutes. Samples were quickly washed 3 times in PBS, and glass cover slips were mounted using ProLong Gold Antifade Mountant (Thermo Fisher Scientific). After 48 hours of drying while protected from light, slides were imaged using a fluorescence microscope at 200× original magnification (Leica, DFC365FX).

### ChIP–quantitative PCR.

The procedure for ChIP–quantitative PCR (ChIP-qPCR) has been described previously and was performed with some modifications (20). Briefly, 24 hours after UVB irradiation, cells were cross-linked with 1% formaldehyde for 10 minutes and subsequently quenched with 0.125 M glycine. Cells were lysed in cell lysis buffer (50 mM HEPES, pH 7.4, 1 mM EDTA, 85 mM KCl, 10% glycerol, 0.5% Nonidet P-40, protease inhibitor) and centrifuged at 1200*g* at 4°C for 5 minutes. After suspension in nucleus lysis buffer (50 mM Tris HCl, pH 8.0, 2 mM EDTA, 150 mM NaCl, 5% glycerol, 1% Triton X-100, 1% SDS, protease inhibitor), nuclear pellets were sonicated with Covaris S2 for 24 minutes at the recommended manufacturer settings. Shearing was validated by BioAnalyzer (Agilent). Samples were diluted 10-fold with ChIP dilution buffer and were immunoprecipitated by adding anti-AhR Ab, anti-SP1 Ab, or normal rabbit IgG overnight at 4°C. Samples were subsequently incubated with Dynabeads Protein G under rotation for 1 hour at room temperature (RT). Beads were washed twice with ChIP wash buffer 1 (20 mM Tris HCl, pH 8.0, 150 mM NaCl, 1% Triton X-100, 0.1% SDS, 2 mM EDTA), twice with ChIP wash buffer 2 (20 mM Tris HCl, pH 8.0, 500 mM NaCl, 1% Triton X-100, 0.1% SDS, 2 mM EDTA), and once with ChIP wash buffer 3 (20 mM Tris HCl, pH 8.0, 150 mM NaCl, 500 mM LiCl, 1% Nonidet P-40, 1% deoxycholate, 1 mM EDTA). After washing once in Tris-EDTA buffer (Invitrogen) (10 mM Tris HCl, pH 8.0, 1 mM EDTA), bound DNA was eluted from beads using a ChIP Elute Kit (Takara) and analyzed with RT-qPCR with primers flanking the putative SP1 binding site in the promoter regions of MMP genes (Supplemental Table 1). Ct values were normalized to 5% input and corresponding nonirradiated samples to calculate fold enrichment.

### Cloning of luciferase constructs.

Oligos containing 1000 bp of human MMP2 and MMP11 promoter regions of human MMP2 with flanking digestion sites for XhoI and HindIII were synthesized by Thermo Fisher Scientific (GeneArt). For mutant oligos, 2 putative SP1 binding sites were abrogated for each promoter (MMP2: –104, –93; MMP11: –65, –43), as previously described (21, 22). After sequencing was verified by the manufacturer, oligos and the firefly luciferase vector pGL4.11(luc2p) (Promega) were digested overnight at 37°C by XhoI and HindIII (New England Biosciences) and separated by agarose gel. Corresponding bands were cut out and purified by gel purification kit (Qiagen) and ligated with T4 DNA ligase (New England Biosciences). Competent bacteria were transformed, plated on ampicillin-containing agar plates, and incubated overnight at 37°C. After inoculation of luria broth, cultures containing ampicillin, plasmid was extracted by QIAprep Spin MiniPrep kit (Qiagen) and quantified by NanoDrop. Successful cloning was verified by diagnostic digest separated by 1% agarose gel.

### Dual luciferase assay.

Serum starved cells were cotransfected with pGL4.75 CMV-Ren (Promega) and firefly luciferase constructs containing MMP promoters at a 9:1 ratio. A total of 24 hours after irradiation, the Dual-Glo Luciferase kit was used to measure relative promoter activities. Briefly, firefly luciferase reagent was added directly to media and incubated for 5 minutes at RT. The supernatant was then transferred to a white 96-well plate (Corning), and luminescence was quantified by a plate reader. Renilla luciferase reagent was subsequently added, and luminescence was quantified again after 5 minutes of incubation. RLUs were calculated by normalizing firefly signals with renilla signals, and fold change was calculated by normalizing with nonirradiated samples.

### Western blot.

Biological replicates were pooled and lysed in RIPA buffer supplemented with cOmplete protease inhibitor cocktail (Sigma) and PhosSTOP phosphatase inhibitor (Sigma) for 45 minutes on a rotator at 4^o^C. After spinning at 15,000 rpm for 15 minutes at 4°C, protein lysate was quantified using bicinchoninic acid protein kit and boiled in sample buffer at 70°C for 10 minutes. Samples were run in Bolt 4%–12% Bis-Tris gels (Thermo Fisher Scientific) according to manufacturer recommendations and transferred to PVDF membranes using the Trans-Blot Turbo system (Bio-Rad). Membranes were subsequently blocked in 5% milk (in TBST) for 1 hour at RT and incubated with primary antibody diluted in 5% BSA (in TBST) overnight at 4°C. After 3 washes in TBST, membranes were incubated with a secondary antibody diluted in 5% milk (in TBST) for 1 hour at RT. After another set of 3 washes in TBST, membranes were developed using SuperSignal West Pico Plus chemiluminescence kit (Thermo Fisher Scientific) and imaged with ChemiDoc (Bio-Rad).

### RT-qPCR.

RNA was extracted using RNeasy Plus kit (Qiagen), and cDNA was subsequently synthesized using iScript (Bio-Rad). Next, RT-qPCR was performed on diluted cDNA samples using iTaq master mixes (Bio-Rad) and the primers listed in Supplemental Table 1, which were designed using PrimerBlast. Ct values for genes of interest were first normalized to hypoxanthine-guanine phosphoribosyltransferase values and then to the control samples to calculate relative fold change.

### Statistics.

Data were analyzed with Pearson’s correlation, unpaired 2-tailed Student’s *t* test, Mann-Whitney *U* test, 1-way ANOVA, or 2-way ANOVA in GraphPad Prism, as indicated in the corresponding legends. A *P* value less than 0.05 was considered statistically significant.

### Study approval.

Animal experiments were conducted under protocols approved by the Animal Use and Care Committee at Johns Hopkins University (MO18M329).

## Author contributions

DJK conceived the original idea and conducted all experiments. DJK, AI, ALC, and SK designed the experiments, analyzed data, and wrote the paper. ALC and SK provided financial support for the project.

## Supplementary Material

Supplemental data

## Version 1 03/22/2022

In-Press Preview

## Version 2 05/09/2022

Electronic publication
