# Supplementary material for: UVB-mediated DNA damage induces matrix metalloproteinases to promote photoaging in an AhR- and SP1-dependent manner
Source: JCI Insight. 2022 May 9;7(9):e156344. doi: 10.1172/jci.insight.156344 (PMC9090247; doi:10.1172/jci.insight.156344)
Supplement: Supplemental data [file jciinsight-7-156344-s183.pdf]

Supplemental Data

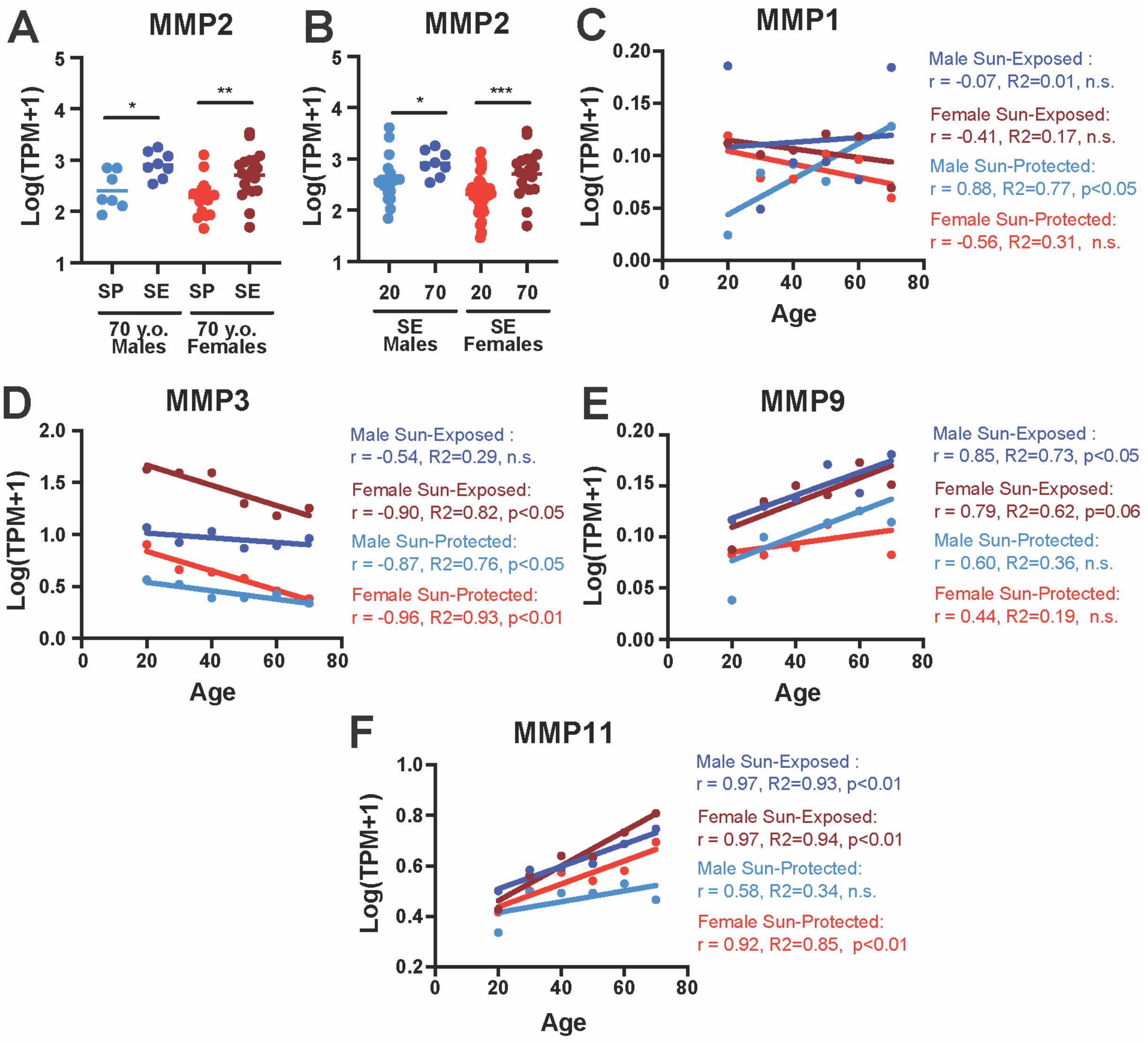

Supplemental Figure 1: MMP genes have distinct expression profiles in human skin across age and differentially correlate with AhR pathway. (A-B) *Mmp2* mRNA expression in human skin from (A) donors in their 70s and (B) sun-exposed samples. (C-F) Average mRNA expression of (C) *Mmp1*, (D) *Mmp3*, (E) *Mmp9*, and (F) *Mmp11* with increasing age in human skin, stratified by sex and sun exposure. All data are $\log_{10}$ transformed TPM values from GTEx. n=234 male sun-exposed samples, 467 female sun-exposed samples, 193 male sun-protected samples, 411 female sun-protected samples. P values were calculated by Pearson's correlation, unpaired two-tailed Mann-Whitney test, or one-way ANOVA. * P < 0.05, ** P < 0.01, *** P < 0.001

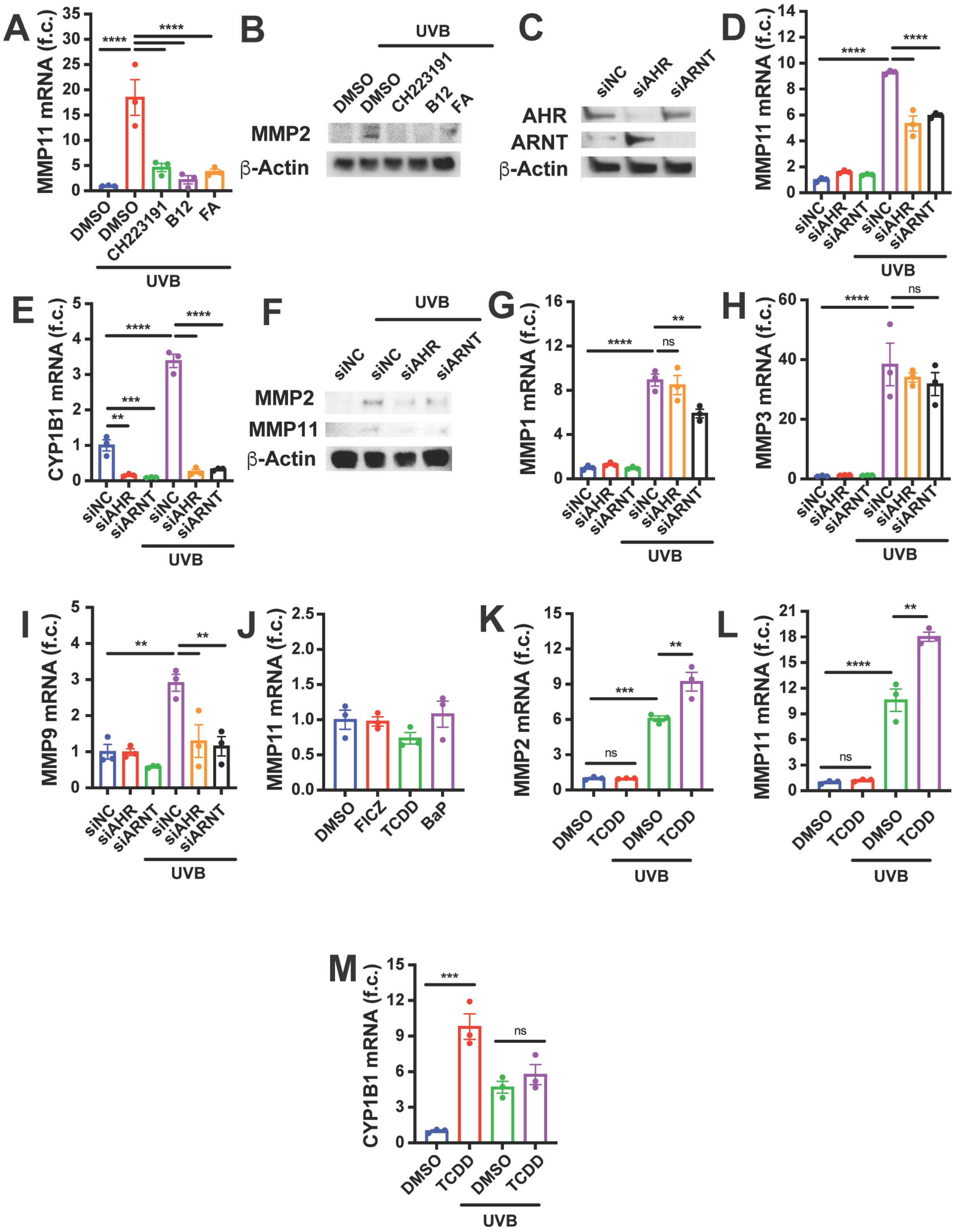
A
MMP11 mRNA (f.c.)
DMSO
DMSO
CH223191
B12
FA
UVB
B
UVB
DMSO
DMSO
CH223191
B12
FA
MMP2
β-Actin
C
siNC
siAHR
siARNT
AHR
ARNT
β-Actin
D
MMP11 mRNA (f.c.)
siNC
siAHR
siARNT
siNC
siAHR
siARNT
UVB
E
CYP1B1 mRNA (f.c.)
UVB
F
UVB
siNC
siNC
siAHR
siARNT
MMP2
MMP11
β-Actin
G
MMP1 mRNA (f.c.)
ns
UVB
H
MMP3 mRNA (f.c.)
ns
UVB
I
MMP9 mRNA (f.c.)
UVB
J
MMP11 mRNA (f.c.)
DMSO
FICZ
TCDD
BaP
K
MMP2 mRNA (f.c.)
ns
DMSO
TCDD
DMSO
TCDD
UVB
L
MMP11 mRNA (f.c.)
ns
UVB
M
CYP1B1 mRNA (f.c.)
ns
DMSO
TCDD
DMSO
TCDD
UVB

Supplemental Figure 2: Modulation of AhR activity has distinct effects on MMP gene expression. (A) Same experiment as Fig. 2A-C. *Mmp11* mRNA expression was measured by RT-qPCR and normalized to *Hprt1* and DMSO-treated sample (n=3). (B) Normal human dermal fibroblasts (NHDFs) were irradiated with 200 mJ/cm^2 UVB and subsequently treated with DMSO, 10 uM CH-223191, 50 ng/mL vitamin B12, or 20 ng/mL folic acid (FA) for 24 h. Protein levels of MMP2 and β-Actin were measured by western blot. (C-I) Same experiment as Fig. 2D. (C) Protein levels of AHR, ARNT, β-Actin were measured by western blot. (D) *Mmp11*, (E) *Cyp1b1*, (G) *Mmp1*, (H) *Mmp3*, and (I) *Mmp9* mRNA was measured by RT-qPCR and normalized to *Hprt1* and siNC-transfected samples (n=3). (F) Protein levels of MMP2, MMP11, and β-Actin were measured by western blot. (J) Same experiment as Fig. 2E-F. *Mmp11* mRNA was measured by RT-qPCR and normalized to *Hprt1* and DMSO-treated sample (n=3). (K-M) HaCaT keratinocytes were irradiated with UVB and subsequently treated with DMSO or 25 nM TCDD for 24 h. (E) *Mmp2*, (F) *Mmp11*, and (G) *Cyp1b1* mRNA was measured by RT-qPCR and normalized to *Hprt1* and DMSO-treated samples (n=3). Data are means ± SEM. P values were calculated by one-way ANOVA. * $P < 0.05$, ** $P < 0.01$, **** $P < 0.0001$

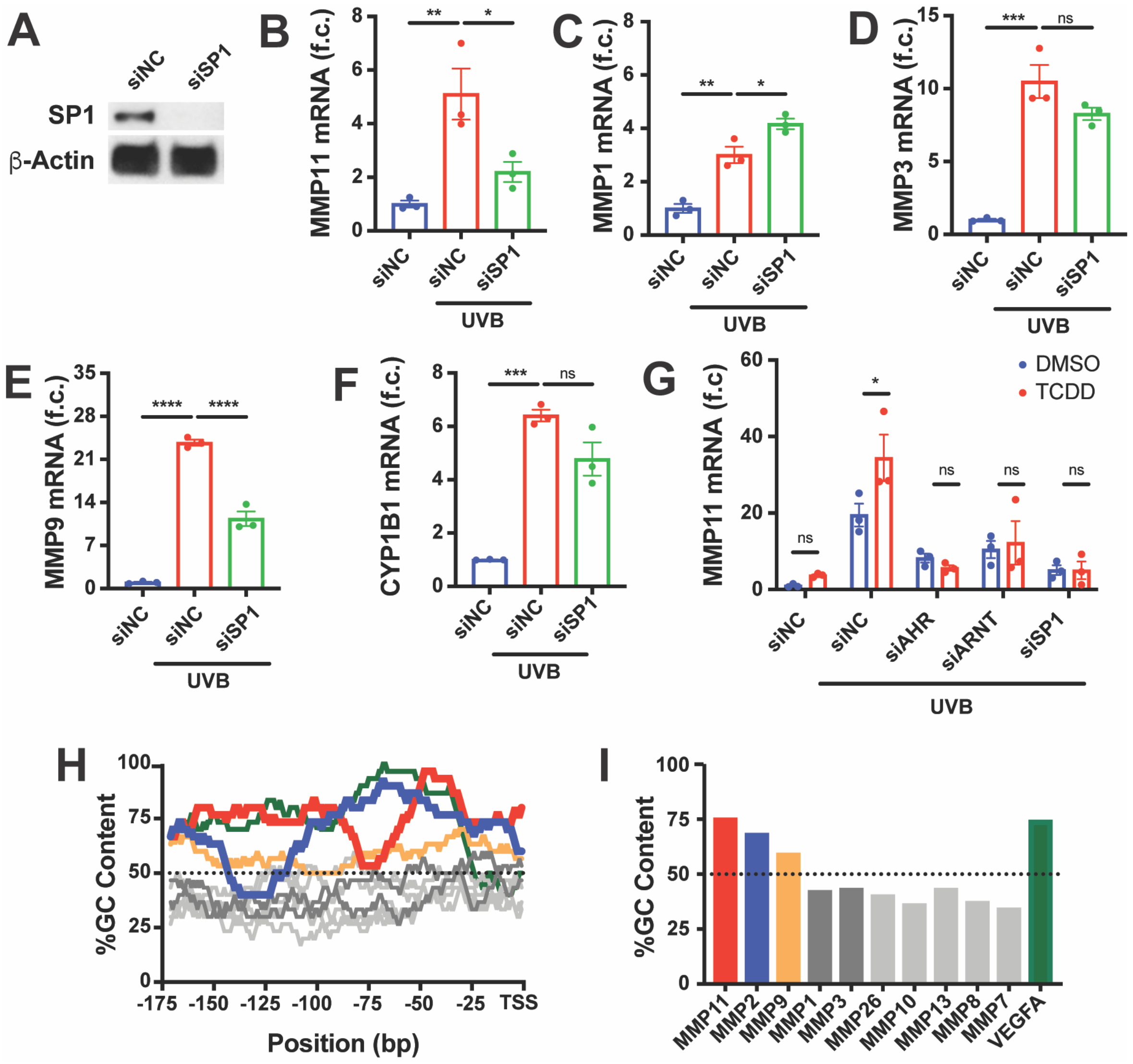

Supplemental Figure 3: MMPs are differentially affected by SP1 knockdown and feature consistent GC content in their proximal promoters. (A-F) Same experiment as Fig. 3A-B. (A) Protein levels of SP1 and β-Actin were measured by western blot. (B) *Mmp11*, (C) *Mmp1*, (D) *Mmp3*, (E) *Mmp9*, and (F) *Cyp1b1* mRNA was measured by RT-qPCR and normalized to *Hprt1* and siNC-transfected samples (n=3). (G) Same experiment as Fig. 3C. *Mmp11* mRNA was measured by RT-qPCR and normalized to *Hprt1* (n=3). Data are means ± SEM. P values were calculated by one-way or two-way ANOVA. * $P < 0.05$, *** $P < 0.001$, **** $P < 0.0001$. (H-I) Proximal promoter regions (200 bp upstream of transcriptional start sites) of MMP genes and *Vegfa* were analyzed for % GC content. (H) GC content from a 30 bp sliding window, traversing from -170 bp to TSS of promoters. (I) GC content of entire 200 bp region of promoters.

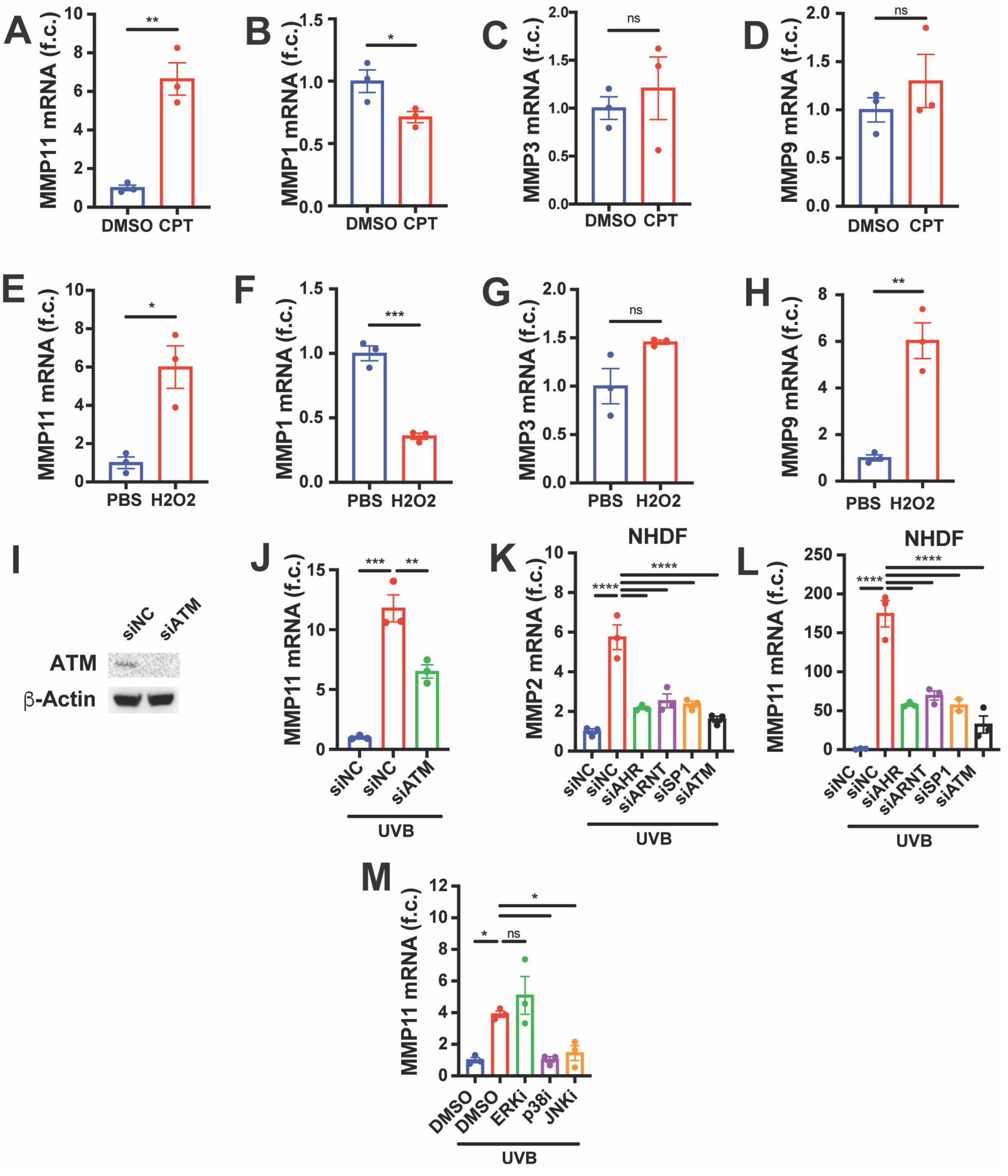

Supplemental Figure 4: DNA damage differentially modulates MMP mRNA expression, and ATM is required for *Mmp2* and *Mmp11* mRNA expression in dermal fibroblasts. (A-D) Same experiment as Fig. 4A. (A) *Mmp11*, (B) *Mmp1*, (C) *Mmp3*, and (D) *Mmp9* mRNA was measured by RT-qPCR and normalized to *Hprt1* and DMSO-treated samples (n=3). (E-H) Same experiment as Fig. 4B. (E) *Mmp11*, (F) *Mmp1*, (G) *Mmp3*, and (H) *Mmp9* mRNA was measured by RT-qPCR and normalized to *Hprt1* and PBS-treated samples (n=3). (I-J) Same experiment as

Fig. 4D,F. (I) Protein levels of ATM and β-Actin were measured by western blot. (J) *Mmp11* mRNA was measured by RT-qPCR and normalized to *Hprt1* and siNC-transfected samples (n=3). (K-L) NHDF cells were transfected with indicated siRNA, irradiated with 200 mJ/cm^2 UVB, and lysed for analysis 24 h later. (K) *Mmp2* and (L) *Mmp11* were measured by RT-qPCR and normalized to *Hprt1* and siNC-transfected samples (n=3). (M) Same experiment as Fig. 4E. *Mmp11* mRNA was measured by RT-qPCR and normalized to *Hprt1* and DMSO-treated samples (n=3). Data are means ± SEM. P values were calculated by unpaired two-tailed Student‘s t-test or one-way ANOVA. * $P < 0.05$, ** $P < 0.01$, *** $P < 0.001$, **** $P < 0.0001$

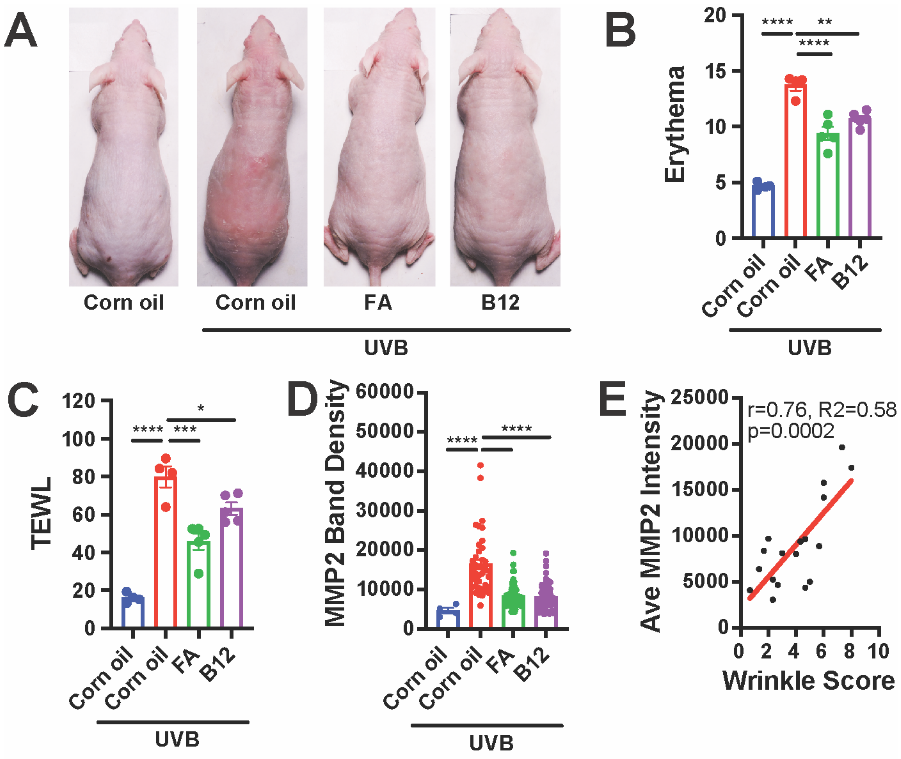

Supplemental Figure 5: UV-induced erythema, TEWL, and MMP2 expression are decreased with B12 and FA treatment and MMP2 expression correlates with wrinkle severity. Same experiment as Figure 5. (A-C) At week 10, mice were evaluated for sunburns. (A) Representative images of sunburns from each treatment arm. (B) Erythema and (C) transepidermal water loss (TEWL) were quantified by skin probe. (D) Intensity of MMP2 expression by bands in fluorescent IHC samples. Data are means ± SEM. (E) Correlation between average MMP2 band intensity and corresponding wrinkle score for each mouse. P values were calculated by one-way ANOVA or Pearson's correlation. * $P < 0.05$, ** $P < 0.01$, *** $P < 0.001$, **** $P < 0.0001$

Gene	Forward primer	Reverse primer
Hprt1	5’-CCTGGCGTCGTGATTAGTGAT-3’	5’-AGACGTTCAGTCCTGTCCATAA-3’
Cyp1b1	5’-TCCTCCTCTTCACCAGGTATCC-3’	5’-CCAGGACATAGGGCAGGTTG-3’
Mmp2	5’-CGATGGATACCCCTTTGACGG-3’	5’-CCATACTTCACACGGACCACTTG-3’
Mmp11	5’-CCAGGATGCTGATGGCTATGC-3’	5’-AGGAAAGTGTTGGCAGGCTC-3’
Mmp1	5’-ATGCTGAAACCCTGAAGGTG-3’	5’-GAGCATCCCCTCCAATACCT-3’
Mmp3	5’-GTCTCTTTCACTCAGCCAAC-3’	5’-ATCAGGATTTCTCCCCTCAG-3’
Mmp9	5’- GGCAGCTGGCAGAGGAATAC-3’	5’-GGCCCCAGAGATTTCGACTC-3’
ChIP *Mmp2*	5’-ACGAGGTCGTGCACTGAG-3’	5’-GAAACAAGGGAGGGCAGC-3’
ChIP *Mmp11*	5’-CGGCTGCTAGGAGAGTTCAG-3’	5’-AGCAGCAGCAGCAGCATC-3’
ChIP *Mmp1*	5’-CAGGCAGCTTAACAAAGGCAG-3’	5’-CGCACCTGATGGCTGTTC-3’
ChIP *Mmp3*	5’-GAACCAGCAAATCCAACG-3’	5’-GAGAGAAGAAGTAGGTTGACTTGG-3’
ChIP *Mmp9*	5’-TCTCATGCTGGTGCTGCC-3’	5’-CTTTAAGGAGGCGCTCCTGTG-3’

Supplemental Table 1: RT-qPCR primers for human genes
